# Internal Zn allocation influences Zn deficiency tolerance and grain Zn loading in rice (*Oryza sativa* L.)

**DOI:** 10.3389/fpls.2013.00534

**Published:** 2013-12-24

**Authors:** Somayanda M. Impa, Anja Gramlich, Susan Tandy, Rainer Schulin, Emmanuel Frossard, Sarah E. Johnson-Beebout

**Affiliations:** ^1^Crop and Environmental Sciences Division, International Rice Research InstituteMetro Manila, Philippines; ^2^Department of Environmental System Science, Institute of Terrestrial EcosystemsETH Zürich, Switzerland; ^3^Department of Environmental System Science, Institute of Agricultural SciencesETH Zürich, Switzerland

**Keywords:** rice, continued root uptake, grain Zn, grain Zn loading, Zn biofortification, Zn deficiency tolerance, Zn remobilization

## Abstract

One of the important factors that influences Zn deficiency tolerance and grain Zn loading in crops is the within-plant allocation of Zn. Three independent experiments were carried out to understand the internal Zn distribution patterns in rice genotypes grown in Zn-sufficient and Zn-deficient agar nutrient solution (ANS). In one of the experiments, two rice genotypes (IR55179 and KP) contrasting in Zn deficiency tolerance were leaf-labeled with ^65^Zn. In the other two experiments, two Zn biofortification breeding lines (IR69428 and SWHOO) were either root- or leaf-labeled with ^65^Zn. Rice genotype IR55179 showed significantly higher Zn deficiency tolerance than KP at 21 and 42 days after planting. When KP was Zn-deficient, it failed to translocate ^65^Zn from the labeled leaf to newly emerging leaves. Similarly, the root-to-shoot translocation of unlabeled Zn was lower in KP than in IR55179. These results suggest that some Zn-efficient rice genotypes have greater ability to translocate Zn from older to actively growing tissues than genotypes sensitive to Zn deficiency. Among the two Zn biofortication breeding lines that were leaf-labeled with ^65^Zn at 10 days before panicle initiation stage, ^65^Zn distribution in the grains at maturity was similar between both genotypes in Zn-sufficient conditions. However, under Zn-deficient conditions, SWHOO accumulated significantly higher ^65^Zn in grains than IR69428, indicating that SWHOO is a better remobilizer than IR69428. When the roots of these two Zn biofortication breeding lines were exposed to ^65^Zn solution at 10 days after flowering, IR69428 showed higher root uptake of ^65^Zn than SWHOO in Zn-sufficient conditions, but ^65^Zn allocation in the aerial parts of the plant was similar between both genotypes.

## Introduction

Enriching brown rice Zn concentration to the target of 30 mg kg^−1^ set by the HarvestPlus program would provide 40% of the estimated average requirement for preschool children and non-pregnant and non-lactating women (Saltzman et al., 2013). Achieving the target grain Zn concentration can be particularly challenging under conditions of low available soil Zn, which often occur with reduced conditions developed after flooding in paddy fields (Johnson-Beebout et al., 2009). Therefore, understanding the plant uptake, translocation, and loading of Zn to rice grains is crucial, as this influences not only grain Zn but also the ability of a plant to grow and yield in Zn-deficient soil. Zn deficiency tolerance (Zn-efficiency) of a rice genotype is known to be influenced by several root- and shoot-related processes such as higher root uptake of Zn, high root-to-shoot translocation of Zn, biochemical use of Zn, subcellular compartmentation and enhanced translocation of Zn from older to new tissues under Zn-deficient conditions (Hacisalihoglu and Kochian, 2003; Impa and Johnson-Beebout, 2012). Higher Zn uptake is often strongly related to Zn deficiency tolerance and is known to be influenced by several root related processes, such as efflux of phytosiderophores and low molecular weight organic acids, proton exudation, mycorhizal colonization, and formation of iron plaques on roots (Graham and Rengel, 1993; Rose et al., 2013). The effectiveness of each of these mechanisms is likely to vary depending on the soil environment, genotype, and Zn status (Impa and Johnson-Beebout, 2012).

Accumulation of Zn in rice grains can occur through continued root uptake during grain filling stage and/or through remobilization of earlier taken up and stored Zn from sources tissues to grain. Unlike wheat, in rice the presence of continuous xylem stream implies that remobilization of Zn may not contribute significantly to grain Zn accumulation, provided there is sufficient Zn supply to roots (Stomph et al., 2009). Jiang et al. (2007) found that in aerobic rice genotypes grown in Zn sufficient nutrient solution continued root uptake was the predominant source of grain Zn accumulation. Contrastingly, according to Wu et al. (2010) in a high grain Zn rice genotype most of the Zn accumulated in grain was through remobilization from sources tissues rather than continued root uptake during grain filling. Moreover, in rice Zn was found to be supplied directly via phloem to grains and husks (Yoneyama et al., 2010). Our previous results using a set of rice genotypes contrasting for grain Zn, indicated that under sufficient Zn supply at the grain-filling stage, plants predominantly take up Zn through roots and transport it toward grain (Impa et al., 2013). Remobilization of Zn from source tissues to grain usually occurs under either Zn-deficient conditions or in plants with an accelerated rate of senescence or high phloem mobility for Zn (Bukovac and Wittwer, 1957; Kochian, 1991; Sperotto et al., 2009; Impa et al., 2013). Moreover several transporters involved in enhancing grain Zn accumulation in rice through either of these sources have been identified (Sperotto et al., 2009, 2010; Johnson et al., 2011; Lee et al., 2011; Bashir et al., 2012). Recently, Sperotto (2013), proposed a model for grain Zn loading according to which under Zn sufficient condition, grain Zn accumulation in rice occurs mainly through continued root uptake during grain filling stage with very little contribution from remobilization of Zn from stem and flag/upper leaf reserves. Whereas, under Zn deficient condition both continued root uptake and remobilization of Zn from source tissues contribute equally to grain Zn loading.

The identification of the predominant modes of grain Zn loading in rice genotypes would help breeders not only to identify donors for targeted Zn biofortification breeding but also to optimize the time and method of Zn fertilizer application. If a genotype predominantly depends on continued root uptake for grain Zn loading, it would be important to make soil Zn more available at later growth stages through Zn fertilization combined with terminal drying prior to harvest in flooded paddy fields. If a genotype has remobilization of Zn from source tissues as the main source of grain Zn loading, it would be important to get sufficient Zn into the plant early in the season through either soil Zn fertilization during the vegetative stage or foliar Zn application at heading or the early grain-filling stage (Boonchuay et al., 2013; Mabesa et al., 2013). Genotypes with both efficient root uptake of Zn during grain filling and remobilization of Zn from source tissues would be the most preferred ones for enriching grain Zn, because they would be expected to perform better across a variety of environments in which soil Zn can be available at different times of the season.

From our previous study, it was predicted through mass balance calculations that under Zn-sufficient conditions continued root uptake during grain filling was the predominant source of grain Zn loading in most rice genotypes, whereas, in Zn-deficient conditions, genotypes varied in their predominant source of grain Zn loading, with some genotypes showing continued root uptake as the predominant source while in others remobilization was predominant (Impa et al., 2013). One of the major limitations in predicting the predominant grain Zn loading sources through mass balance calculation is the difficulty in tracking the actual movement of Zn within different plant tissues. So, in the present study, two Zn biofortification breeding lines with high grain-Zn, namely, SWHOO and IR69428, contrasting in their sources of grain Zn loading as predicted from mass balance data, were selected for tracking the actual movement of Zn using ^65^Zn labeling to either leaves or roots. In addition, two other genotypes contrasting in Zn deficiency tolerance, namely, KP and IR55179, were selected to assess the movement of Zn from older leaves to new leaves at the vegetative stage.

The two specific objectives investigated in the present study were (1) to evaluate the effect of remobilization of Zn from old to new leaves on Zn deficiency tolerance and (2) to determine the predominant source of grain Zn loading (remobilization vs. continued root uptake) in high-grain-Zn genotypes. In particular, the following hypotheses were tested: (H1) Remobilization of Zn from old to new leaves would be higher in a Zn-deficiency-tolerant genotype (IR55179) than in a Zn-deficiency-sensitive genotype (KP) in Zn-deficient conditions. (H2.1) In some high-grain-Zn genotypes, remobilization of Zn from leaves could be the major source of Zn to grains whether in Zn-deficient or Zn-sufficient conditions. (H2.2) Less Zn remobilization from leaves to grain during grain filling in some of the high-grain-Zn genotypes is associated with continued Zn uptake through roots under Zn-sufficient conditions.

## Materials and methods

### Plant material

The seeds were obtained from the Plant Breeding, Genetics, and Biotechnology (PBGB) Division of the International Rice Research Institute (IRRI), Philippines. The full names of the genotypes, their designations and their descriptions are given in Table 1.

**Table 1:** **Initial seed Zn concentration (mg kg^−1^) and descriptions of the rice genotypes used in the experiments**.

**Genotype name**	**Designation**	**Seed Zn concentration **before sowing (mg kg^−1^**)**	**Description**
SWHOO	SWHOO	30	Zn biofortification donor
IR69428-6-1-1-3-3 (IR68150 × IR65600-1-3-2)	IR69428	23	Zn biofortification breeding line
IR55179-3B-11-3 (IR4630-22-2-5-1-3 × Nona Bokra)	IR55179	18	Tolerant of Zn deficiency
Kinandang Patong	KP	20	Sensitive to Zn deficiency

Seed includes hull and brown rice. Parents of breeding lines are given in parentheses.

### Plant growth

The experiments were carried out in the Eidgenössische Technische Hochschule (ETH) growth chamber facility, at Eschikon, near Zürich, Switzerland. The plants were grown in an 11 h/13 h day/night cycle at a temperature of 30 and 23°C and relative humidity of 60 and 70% during day and night, respectively. All the plasticware and glassware were thoroughly washed with soap solution in a dishwasher, soaked in 10% HNO_3_ for 2 h and then rinsed twice with deionized water to make them Zn-free prior to the experiment. Initially, the seeds were kept for germination on a moist filter paper in petri dishes kept in the dark at 23 ± 1°C for 5 days. The sprouted seeds were floated on 0.5 mM CaCl_2_ solution with 10 μM iron sodium ethylene-diamine-tetra-acetate (FeNaEDTA) for 1 week. The seedlings were then transferred to pots filled with half-strength modified Yoshida nutrient solution (YNS) without Zn for 1 week and later on transferred to half-strength YNS with the respective Zn treatments for a week. The composition of modified YNS at full strength is as follows: 1.77 mM NH_4_NO_3_, 0.32 mM NaH_2_PO_4_ · 2H_2_O, 0.5 mM K_2_SO_4_, 1 mM CaCl_2_ · 2H_2_O, 1 mM MgSO_4_ · 7H_2_O, 9 μM MnCl_2_ · 4H_2_O, 0.5 μM (NH_4_)_6_Mo_7_O_24_ · 4H_2_O, 18.5 μM H_3_BO_3_, 0.16 μM CuSO_4_ · 5H_2_O, 36 μM FeNaEDTA (Impa et al., 2013). Zn was supplied at 0.005 μM and 1.5 μM ZnSO_4_ · 7H_2_O to establish Zn-deficient and Zn-sufficient conditions, respectively. The pots were replenished with fresh YNS once every 3 or 4 days. Further, the seedlings were transferred to agar nutrient solution (ANS) containing 0.1% agar in modified full-strength YNS with Zn supplied to establish Zn-deficient and Zn-sufficient conditions. The day when the plants were first transferred to ANS medium is considered as day 0 (0 days after planting in ANS) for all the reported data. The pH of the ANS was adjusted to 8 with NaOH. The pots were arranged in a randomized complete block design and ANS in these pots was replenished once every 14 days. The lids of the 10-L-capacity plastic pots used in the experiment had eight openings to fit the plants.

### Experiment-1

Two rice genotypes, IR55179 (Zn-deficiency-tolerant) and KP (Zn-deficiency-sensitive), were grown in ANS with sufficient and deficient Zn as described above. Before leaf labeling with ^65^Zn, 10 plants or replications from each genotype were destructively sampled to estimate the total biomass and Zn concentrations in different plant tissues. Leaf labeling was done at 21 days after growing the plants in ANS (DAP), wherein the top fully expanded leaf on main tiller was dipped in 5 mL of 600 kBq ^65^Zn solution in an Eppendorf tube for 10 s. The ^65^Zn solution contained around 13.5 μ Ci ^65^Zn taken in an eppendorf tube, along with 10% Tween 80 and volume made up to 5 mL using sterile water. The same procedure was repeated the next day for 20 s. Leaf labeling was done in three plants or replications for each treatment and genotype. The plants were harvested at 3 weeks after labeling. Another set of plants with three replications each in Zn-sufficient and Zn-deficient conditions was maintained unlabeled and harvested at 42 DAP. After harvest, the unlabeled plants were oven-dried at 80°C until constant weight was obtained. The dry plant tissue samples were digested in HNO_3_, followed by H_2_O_2_ to extract Zn (Huang and Schulte, 1985), and the digests were analyzed for Zn by inductively coupled plasma-optical emission spectrometry analysis (VISTA-MPX, CCD, and Simultaneous ICP-OES). For the wet digestion of plant tissues, around 200 mg of dry powder of unlabeled plant tissues was weighed in digestion tubes, to which 15 mL of 65% HNO_3_ was added, and the tubes were kept in heating blocks with a temperature of 120°C for 90 min. After this, the digestion tubes were allowed to cool for 30 min under a fume hood and around 3 mL of 30% H_2_O_2_ was added to the digestion tubes. After another cycle of heating for 90 min at 120°C and cooling for 30 min, the digests were analyzed as the acid digests for Zn by ICP-OES. The labeled plants after harvest were oven-dried at 50°C for 3 days, weighed and analyzed for ^65^Zn in roots, stems and leaves using γ-spectrometry (high purity germanium detectors, ORTEC, USA, with adjusted calibration for the geometry for the plant samples).

### Experiment-2

Two high-grain-Zn genotypes (IR69428 and SWHOO) were grown in ANS with sufficient Zn until 30 DAP. Before leaf labeling, initial destructive biomass sampling of 10 plants or replications each from both genotypes was done to estimate the total biomass and Zn concentration in different plant tissues. Leaf labeling was done at 30 DAP (which is approximately 2 weeks before panicle initiation) by dipping the top fully expanded leaf on main tiller for 10 s into an Eppendorf tube filled with 5 mL of 600 kBq ^65^Zn solution. The same procedure was repeated the next day for 20 s. After labeling, a set of plants (three replications for each genotype) was grown in Zn-deficient ANS and another set (three replications for each genotype) in Zn-sufficient ANS until maturity. In addition, three replicate plants per genotype were also maintained unlabeled in each Zn treatment. All the plants were harvested at physiological maturity (around 122 and 143 DAP for SWHOO and IR69428, respectively). The processing of harvested plants for dry weight, Zn concentration, and ^65^Zn distribution was similar to Experiment-1 except that the harvested plants were separated into roots, stems, leaves, rachis, and grains.

### Experiment-3

Two high-grain-Zn genotypes (IR69428 and SWHOO) were grown in ANS with sufficient Zn until the early grain filling stage. Before initiating root labeling with ^65^Zn, initial destructive biomass sampling of five plants or replications each from both genotypes was done to estimate the total biomass and initial Zn concentration in different plant tissues. Three plants or replications from both the genotypes were root labeled with ^65^Zn at the early grain-filling stage, i.e., 10 days after 50% flowering (93 and 123 DAP for SWHOO and IR69428, respectively), during which the plant roots were exposed to 185 kBq ^65^Zn in 500 mL of YNS with 1.5 μM ZnSO_4_ · 7H_2_O for 24 h. After exposure to ^65^Zn for 24 h, the labeled roots were washed with deionized water for 1 min, followed by washing in ice-cold desorption solution containing 100 μM ZnSO_4_ · 7H_2_O for 15 min. The roots were again washed with deionized water for 1 min before putting the plants back in ANS with sufficient Zn until maturity. The plants were always (before and after labeling) maintained under Zn-sufficient conditions so that there was enough Zn for root uptake and to make sure that the contribution of remobilization to grain Zn loading was minimal. In addition, three replicate plants per genotype were also maintained unlabeled until maturity. The plants were harvested at physiological maturity. The processing of harvested plants for dry weight, Zn concentration, and ^65^Zn distribution was similar to Experiment-1 except that the harvested plants were separated into roots, stems, leaves, rachis, and grains. The grains of unlabeled plants were manually dehulled and the Zn concentration in both brown rice and hull was quantified through ICP-OES.

### Statistical analysis

Analysis of variance (ANOVA) was performed using R/aov [R version 2.11.0 (2010-04-22)]. Within a data set, means were compared by the least significant difference (LSD) method. Zn efficiency was calculated by the ratio of shoot dry weight under Zn-deficient conditions to shoot dry weight under Zn-sufficient conditions and was expressed in percentages (Rengel and Graham, 1996). Total Zn content in a specific plant tissue was calculated as the product of the tissue's Zn concentration and its dry weight. Root-to-shoot Zn translocation index was calculated as the ratio of total shoot Zn content to total Zn content per plant (Rengel and Graham, 1996). The percent distribution of ^65^Zn accumulated among different plant parts was calculated excluding the labeled part of the leaf (the part of the leaf that was dipped in 5 mL of 600 kBq ^65^Zn solution) as it retained 80–90% of the total ^65^Zn in plants.

## Results

### Experiment-1

**Figure 1:**
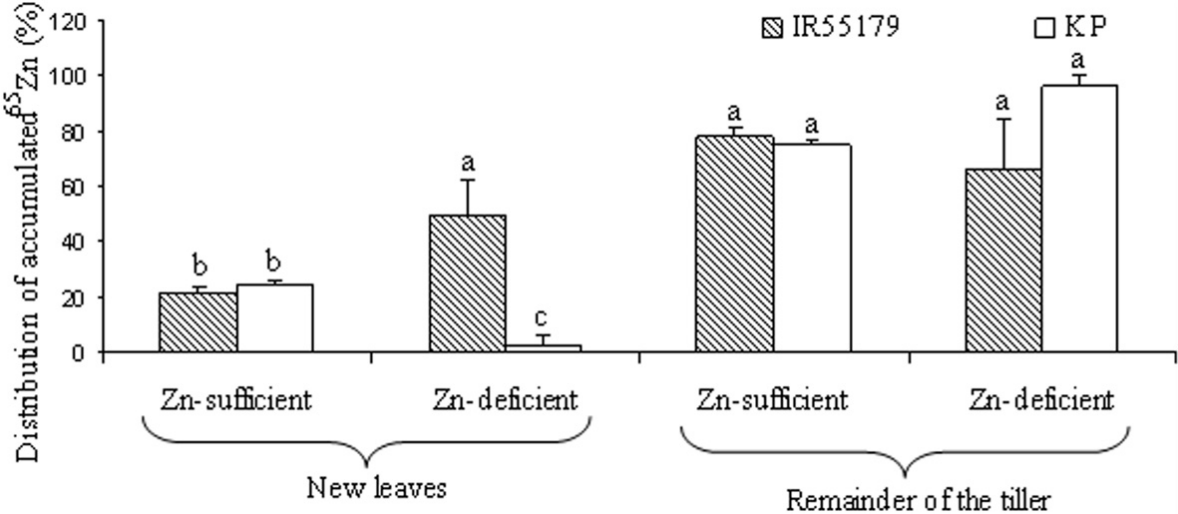
**Distribution of ^65^Zn at 3 weeks after vegetative-stage leaf labeling in different plant parts of rice genotypes grown under Zn-sufficient and Zn-deficient conditions in Experiment-1**. Bars with different letters are significantly different between the two genotypes for a given plant tissue at 5% LSD. Error bars indicate ± SE (*n* = 3).

The relative distribution of accumulated ^65^Zn in new leaves was similar in both genotypes under Zn-sufficient conditions, whereas, under Zn-deficient conditions, IR55179 showed significantly higher accumulation of ^65^Zn than KP (Figure 1). There was no significant difference between the genotypes for percent distribution of accumulated ^65^Zn in the remainder of the labeled tiller in both Zn-sufficient and Zn-deficient conditions (Figure 1). In labeled plants, ^65^Zn activity was seen in new leaves that emerged on the same tiller after labeling and also in the remainder of the labeled tiller but not in other shoots and roots.

**Figure 2:**
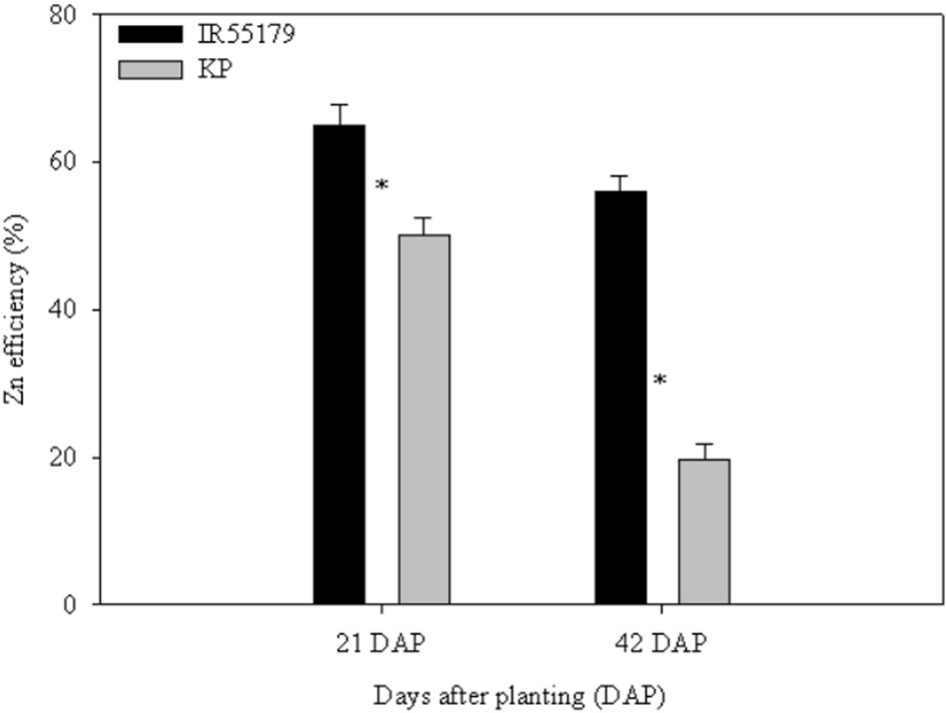
**Zn efficiency (%) of unlabeled rice genotypes at different growth stages in Experiment-1**. Note: “^*^” indicates significant difference between the genotypes within a growth stage at *p* ≤ 0.05. Error bars indicate ± SE (*n* = 10 at 21 DAP and *n* = 3 at 42 DAP). Zn efficiency was calculated by the ratio of shoot dry weight under Zn-deficient conditions to shoot dry weight under Zn-sufficient conditions and is expressed in percentages.

Genotypes differed significantly in their Zn efficiency (indicator of Zn deficiency tolerance) at 21 and 42 DAP in ANS (Figure 2), with IR55179 showing significantly higher Zn efficiency than KP at both growth stages. Zn efficiencies of IR55179 and KP at 21DAP was around 65 ± 2.7 and 50 ± 2.4 respectively and at 41 DAP was around 60 ± 2.3 and 20 ± 2.2 respectively. At 21 DAP, both genotypes showed significantly lower root and shoot Zn concentration and root-to-shoot Zn translocation index in Zn-deficient conditions than in Zn-sufficient conditions (Table 2). At this growth stage, both genotypes showed similar tissue Zn concentration in Zn-deficient conditions, whereas, in Zn-sufficient conditions, IR55179 showed higher root and shoot Zn concentration than KP. At 42 DAP, root Zn concentration in IR55179 did not vary between the Zn treatments, whereas KP showed a significantly lower root Zn concentration in Zn-deficient conditions than in Zn-sufficient conditions (Table 2). Significant treatment differences were noticed for shoot Zn concentration in both genotypes at 42 DAP. In Zn-deficient conditions, IR55179 showed significantly higher root-to-shoot Zn translocation index than KP at 42 DAP.

**Table 2:** **Zn concentrations of different plant parts and root-to-shoot Zn translocation of unlabeled rice genotypes at 21 and 42 days after planting (DAP) under Zn-sufficient and Zn-deficient ANS in Experiment-1**.

**Growth stage**	**Genotype**	**Zn treatment**	**Root Zn concentration **(mg kg^**−1**^)****	**Shoot Zn concentration (mg kg^**−1**^)**	**Root-to-shoot Zn translocation index (%)**
21 DAP	IR55179	Zn-sufficient	52^a^ ± 4.0	76^a^ ± 7.5	77.7^a^ ± 2
		Zn-deficient	26^c^ ± 0.4	11^c^ ± 0.4	52.4^b^ ± 1
	KP	Zn-sufficient	40^b^ ± 2.0	54^b^ ± 1.7	78.9^a^ ± 1
		Zn-deficient	26^c^ ± 0.5	12^c^ ± 0.4	54.0^b^ ± 1
	Genotype		*p* = 0.01	*p* = 0.03	NS
	Treatment		*p* < 0.001	*p* < 0.001	*p* < 0.001
	Genotype × treatment		*p* = 0.015	*p* = 0.003	NS
	5% LSD (Zn × G)		6	10	4.5
42 DAP	IR55179	Zn-sufficient	24.3^a^ ± 1.3	23.1^a^ ± 1	65.8^b, c^ ± 3
		Zn-deficient	23.4^a^ ± 3.4	16.0^b, c^ ± 3	89.0^a^ ± 1
	KP	Zn-sufficient	22.0^a^ ± 1.0	22.0^a, b^ ± 3	73.4^b^ ± 2
		Zn-deficient	17.0^b^ ± 0.3	9.4^c^ ± 1	57.2^c^ ± 4
	Genotype		*p* = 0.009	NS	*p* = 0.03
	Treatment		*p* = 0.03	*p* = 0.002	NS
	Genotype × treatment		NS	NS	*p* = 0.001
	5% LSD (Zn × G)		3.9	6.7	11.5

Zn × G, Zn treatments × genotype. Values with different letters in a column within a growth stage are significantly different at 5% LSD. Shoot includes leaf blade, sheath and stem. The values given are means ± SE (n = 10 for 21 DAP and n = 3 for 42 DAP).

### Experiment-2

**Figure 3:**
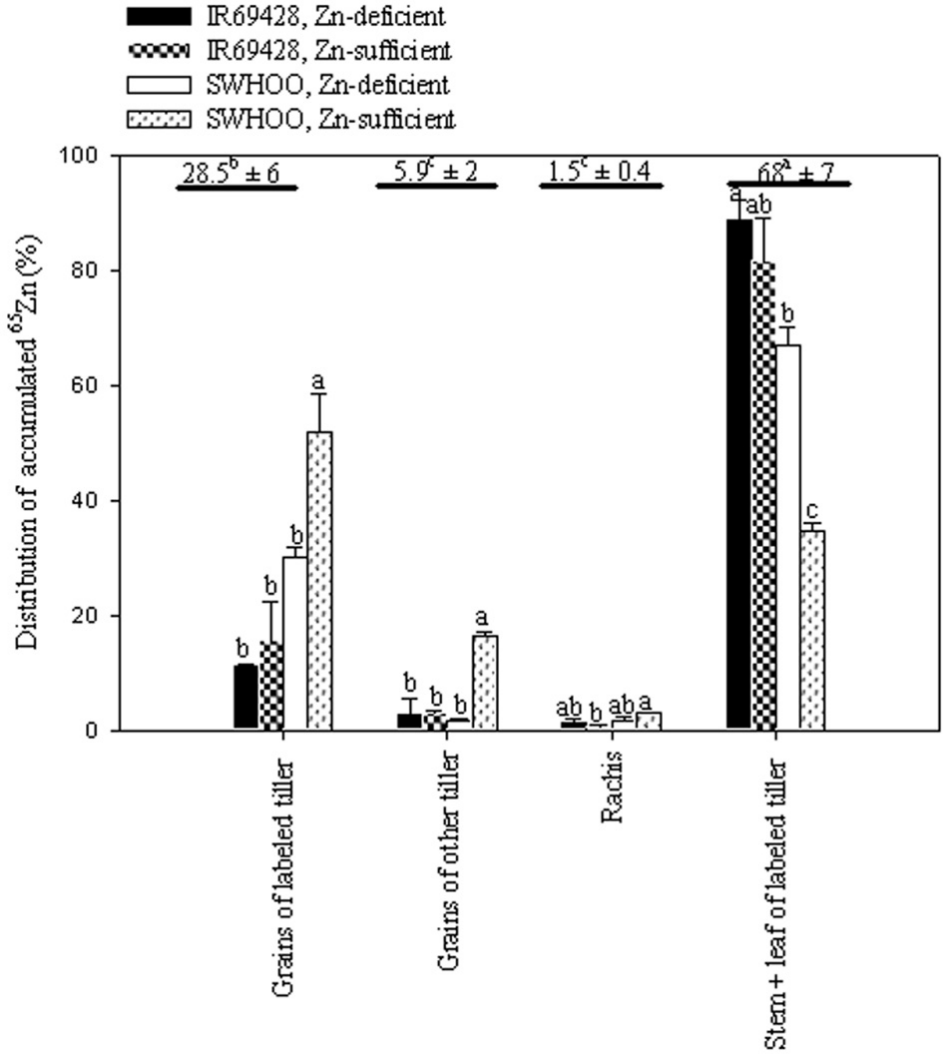
**Distribution of leaf-labeled ^65^Zn among different plant parts measured at maturity in Experiment-2**. Bars with different letters represent significant differences between genotypes for each trait at 5% LSD. The values above the horizontal lines represent the average of relative ^65^Zn accumulation by different plant parts and different letters indicate significant difference at 5% LSD. Grains include brown rice and hull.

Stem + leaves of labeled tillers showed significantly higher percentages of ^65^Zn accumulation than the other plant parts, followed by grains of labeled tillers (Figure 3). The rachis and grains of other tillers showed significantly lower percentages of ^65^Zn accumulation than other plant parts. In both Zn treatments, SWHOO showed higher ^65^Zn accumulation in grains of labeled tillers than IR69428 (Figure 3). SWHOO also showed significantly lower ^65^Zn distribution in shoots of labeled tillers. Percent ^65^Zn accumulation in grains did not differ between the two Zn treatments in IR69428, whereas SWHOO exhibited significantly higher ^65^Zn accumulation in grains under Zn-sufficient conditions than in Zn-deficient conditions. Apart from the leaves, stems, grains, and rachis of labeled tillers, ^65^Zn activity was found in the grains of unlabeled tillers but not in the roots, leaves, and stems of unlabeled tillers. The labeled part of the leaf retained 80–90% of the total ^65^Zn in plants, so it was excluded while calculating the percent ^65^Zn distribution in different parts of the plant.

At 30 DAP, there was no significant difference between the genotypes in root, stem + sheath, and leaf blade Zn concentration in Zn-sufficient conditions (Table 3). At maturity, both genotypes showed significantly lower root, stem + sheath, and leaf blade Zn concentration in Zn-deficient conditions than in Zn-sufficient conditions. Leaf blade Zn concentration at maturity differed significantly between the genotypes in Zn-sufficient conditions, but not in Zn-deficient conditions. IR69428 showed a significantly lower brown rice Zn concentration in Zn-deficient conditions than in Zn-sufficient conditions, whereas brown rice Zn concentration in SWHOO did not differ significantly between the Zn treatments (Table 3). The rachis and hull showed lower Zn concentration than brown rice in both genotypes in both Zn treatments.

**Table 3:** **Zn concentration in different plant parts of unlabeled rice genotypes at different growth stages under Zn-sufficient and Zn-deficient ANS in Experiment-2**.

**Zn concentration in individual plant parts (mg kg^−1^**)								
**Growth stage**	**Genotype**	**Zn treatment**	**Root**	**Stem + sheath**	**Leaf blade**	**Rachis**	**Hull**	**Brown rice**
30 DAP	IR69428	Zn-sufficient	20.8^a^ ± 1	26.7^a^ ± 2	21.5^a^ ± 1.0	–	–	–
	SWHOO		20.7^a^ ± 1	21.9^a^ ± 2	21.5^a^ ± 0.4	–	–	–
	Genotype		NS	NS	NS			
	5% LSD (G)		2.4	4.9	3.4			
Maturity	IR69428	Zn-sufficient	66.2^a^ ± 9	19.5^a^ ± 3	19.0^a^ ± 0.5	11^a^	12^a^	31^a^ ± 1
		Zn-deficient	33.5^b^ ± 6	10.5^b^ ± 0.7	14.5^c^ ± 1	17^a^	8^a^	18^b^ ± 1
	SWHOO	Zn-sufficient	52.4^a, b^ ± 10	19.4^a^ ± 2	13.0^b^ ± 1	17^a^ ± 5	15^a^ ± 3	35^a^ ± 5
		Zn-deficient	31.6^b^ ± 3	10.0^b^ ± 1	12.0^c^ ± 1	8^a^ ± 0.4	7^a^ ± 0.4	28^a, b^ ± 1
	Genotype		NS	NS	*p* < 0.001	NS	NS	*p* = 0.029
	Treatment		*p* = 0.01	NS	*p* = 0.006	NS	NS	*p* = 0.02
	Genotype × treatment		NS	*p* = 0.001	NS	NS	NS	NS
	5% LSD (G × Zn)		26.5	6	2.3	13	13	10

DAP, days after planting in ANS. Values given are averages of 10 replications at 30 DAP and three replications at maturity. Values with different letters are significantly different between genotypes within a growth stage for each trait at 5% LSD (G × Zn, genotype × Zn treatment).

### Experiment-3

**Figure 4:**
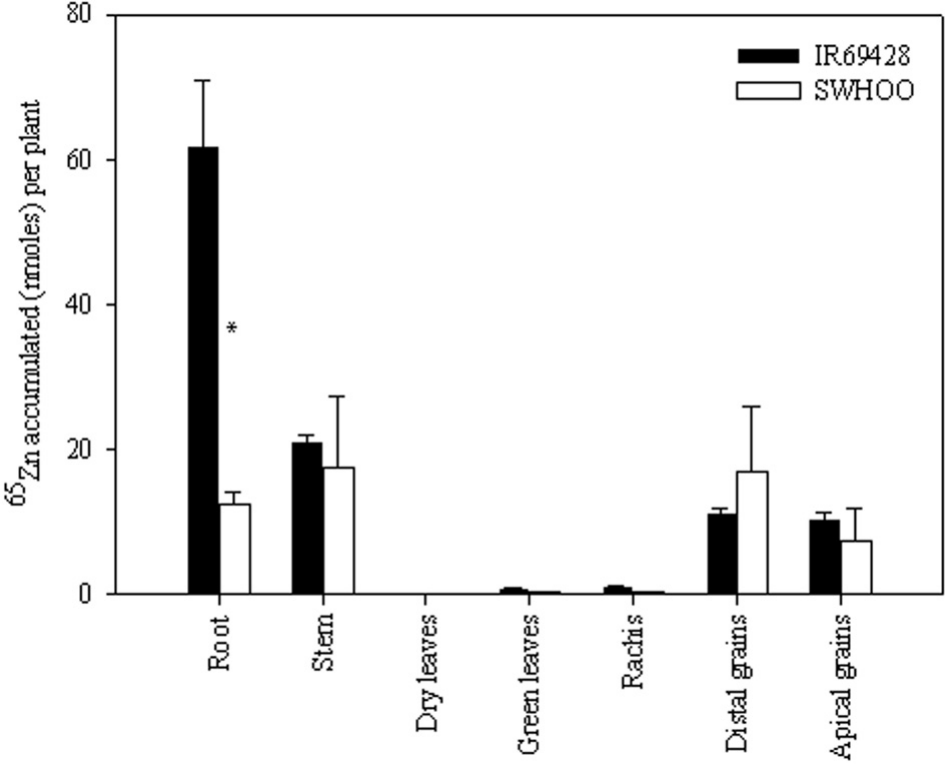
**Distribution of root-labeled ^65^Zn among different plant parts at maturity in Experiment-3**. Note: “^*^” indicates significant difference between the genotypes for a tissue at 5% LSD.

The roots of IR69428 accumulated significantly higher amounts of ^65^Zn than those of SWHOO, but both genotypes accumulated similar amounts of ^65^Zn in the aerial parts, including grains (Figure 4). Dry leaves did not have any ^65^Zn, whereas the green leaves and rachis had very little ^65^Zn in both genotypes (Figure 4). There was no significant difference between the ^65^Zn content of distal and apical grains. In unlabeled plants, both genotypes showed similar root Zn concentration at 10 days after 50% flowering (Table 4). IR69428 showed higher Zn concentration and % Zn allocation in the leaf blade than SWHOO, while the opposite was found in the panicles (Table 4). At maturity, IR69428 showed lower root Zn concentration than SWHOO, which is contrasting to the root Zn concentration between these two genotypes in Experiment-2, but % Zn allocation in roots was similar between both genotypes (Table 4). SWHOO showed lower Zn concentration and % Zn allocation in green leaf blades than IR69428, while the Zn concentration and % Zn allocation in brown rice were higher in SWHOO than in IR69428 at maturity. There was no difference between distal and apical brown rice Zn concentration and % Zn allocation in either of the genotypes (Table 4).

**Table 4:** **Zn concentration and percent Zn allocation in different plant parts of unlabeled plants at different growth stages in Experiment-3**.

**Growth stage**	**Plant parts**	**Zn concentration (mg kg^**−1**^)**		**% Zn allocation in different plant parts**	
		**IR69428**	**SWHOO**	**IR69428**	**SWHOO**
Ten days after 50% flowering	Root	50.3 ± 8	48.8 ± 6	30.6	25.9
	Stem + sheath	20.0 ± 2	26.7 ± 4	45.5	45.9
	Leaf balde	22.5 ± 1	16.6 ± 2	17.2	9.0
	Panicle	30.6 ± 2	41.4 ± 3	6.7	19.0
Maturity	Root	16.6 ± 3.0	30.2 ± 4	12.6	14.0
	Stem + sheath	14.9 ± 0.3	19.6 ± 4	50.7	46.2
	Dry leaves (blade)	19.2 ± 0.5	22.8 ± 3	14.3	4.8
	Green leaves (blade)	30.0 ± 6.0	13.8 ± 1	6.8	3.6
	Rachis	15.4 ± 2.3	19.0 ± 6	0.8	1.0
	Hull	17.5 ± 2.5	14.0 ± 2	1.4	2.7
	Distal brown rice	23.8 ± 1.0	28.3 ± 5	6.2	14.6
	Apical brown rice	23.8 ± 0.5	30.2 ± 5	7.0	13.0

Values are averages of five replications at 10 days after 50% flowering and three replications at maturity.

## Discussion

### Zn deficiency tolerance

KP showed significantly lower Zn efficiency than IR55179 at the vegetative stage, which is consistent with the results of our previous study (Impa et al., 2013). The higher root and shoot Zn concentration of IR55179 than of KP in Zn-sufficient conditions and a similar root and shoot Zn concentration between these genotypes under Zn-deficient conditions at 21 DAP (Table 2) could be due to the overall higher biomass accumulation in KP (2.1 and 9.3 g plant^−1^ at 21 and 42 DAP respectively) than in IR55179 (1.6 and 7.2 g plant^−1^ at 21 and 42 DAP respectively) under Zn-sufficient conditions and a greater reduction in biomass of KP under Zn-deficient conditions than in Zn-sufficient conditions (Table A1), resulting in lower Zn efficiency of KP than of IR55179. Wide variation among rice genotypes in their Zn deficiency tolerance has been noticed in several studies (Quijano-Guerta et al., 2002; Wissuwa et al., 2006; Impa et al., 2013). The higher distribution of ^65^Zn in new leaves of IR55179 than of KP under Zn-deficient conditions indicates a higher remobilization of Zn from labeled leaves to new leaves in IR55179 than in KP (Figure 1), although both genotypes had a similar shoot Zn concentration within a treatment at the time of leaf labeling, i.e., 21 DAP (Table 2). Moreover, KP showed lower remobilization of ^65^Zn from labeled leaves to new emerging leaves under Zn-deficient conditions than in Zn-sufficient conditions. This could be due to the very low Zn in labeled leaves in Zn-deficient conditions and most of the absorbed ^65^Zn is probably strongly bound to cell constituents that could not be remobilized, and similar results were also noticed in wheat by Kutman et al. (2011). Such differences in remobilization of Zn from old leaves to new leaves between the genotypes was not noticed under Zn-sufficient conditions, as there was enough unlabeled Zn in the growth medium for plant uptake and translocation. The inability of KP to remobilize ^65^Zn from labeled leaves to newly emerging leaves could be one of the reasons for its higher sensitivity to Zn deficiency. In IR55179, in addition to higher remobilization of Zn from older to new leaves (H1), a higher root-to-shoot translocation of Zn than in KP (Table 2) resulted in higher tolerance of Zn deficiency.

### Grain Zn loading

#### Remobilization of Zn from source tissues to grain during grain filling

The lower ^65^Zn distribution in shoots and higher distribution in grains of labeled tillers upon leaf labeling with ^65^Zn in SWHOO than in IR69428 (Figure 3) indicates greater remobilization of Zn from stems or leaves to grains in SWHOO than in IR69428 in both Zn treatments. Remobilization of Zn from source tissue to grains was also found to be the predominant source of grain Zn loading in high grain Zn rice genotype IR68144 (Wu et al., 2010). In this experiment, the plants were grown under Zn-sufficient conditions until 30 DAP in ANS and then transferred to Zn-sufficient or Zn-deficient treatments. The similar brown rice Zn concentration between SWHOO and IR69428 in Zn-sufficient conditions and a lower brown rice Zn concentration in IR69428 than in SWHOO under Zn-deficient conditions (Table 3) indicates that, in Zn-deficient conditions, SWHOO was able to remobilize Zn taken up and stored earlier in leaves or tillers when the plants were grown in Zn-sufficient conditions until 2 weeks before panicle initiation. Moreover, SWHOO maintained a similar brown rice Zn concentration between Zn-sufficient and Zn-deficient conditions, unlike IR69428, which showed significantly lower brown rice Zn concentration in Zn-deficient conditions, indicating that IR69428 was unable to efficiently remobilize Zn taken up earlier and stored in source tissues, even when the plants were grown in Zn-deficient conditions. These results suggest that, in SWHOO, remobilization is the predominant source of grain Zn loading (H2.1), but that IR69428 has only limited ability to remobilize Zn from leaves to grains. On the contrary, Jiang et al. (2007) found that in aerobic rice genotypes continued root uptake contributed significantly to grain Zn accumulation and remobilization of Zn from leaves was not that important.

#### Continued root uptake of Zn during grain filling

Both genotypes accumulated ^65^Zn when roots were exposed to ^65^Zn at 10 days after 50% flowering, indicating that the roots continued to take up Zn even after flowering (Figure 4). Similarly, continued root uptake of Zn even after flowering stage has been observed in aerobic rice genotypes irrespective of Zn status of plants (Jiang et al., 2007). Even though both genotypes had similar root dry weight and root length at the time of root labeling (Table A2), IR69428 roots absorbed around 5-fold higher ^65^Zn than SWHOO, indicating a higher root uptake of Zn during the grain-filling period in IR69428 than in SWHOO (H2.2). Most of the Zn taken up by IR69428 accumulated in roots rather than being transported to aerial parts, indicating that, in spite of increased root Zn uptake in IR69428 during grain filling, it did not readily translocate it from roots to shoots. In Experiment-3, we observed contradictory results for root Zn concentration between labeled and unlabeled Zn at maturity. Unlabeled root Zn concentration in IR69428 was lower than in SWHOO (Table 4), whereas ^65^Zn concentration in roots was significantly higher in IR69428 than in SWHOO. And, this difference could be due to the fact that ^65^Zn uptake began only at the grain-filling stage but unlabeled Zn uptake started from the seedling stage. The genotypes were apparently similar in their root Zn uptake at the earlier stage, with the genotype difference appearing only at later growth stages.

In conclusion, Zn-efficient line IR55179 showed significantly higher remobilization of ^65^Zn from older to new leaves and root-to-shoot Zn translocation than KP, suggesting that Zn distribution to active growing parts enhanced Zn efficiency. High-grain-Zn line SWHOO exhibited higher remobilization of Zn from source tissue to grain than IR69428 in both Zn-deficient and Zn-sufficient conditions. This indicates that rice genotypes vary in their phloem mobility of Zn from leaves to grain. There was a higher root uptake of Zn in IR69428 than in SWHOO at the grain-filling stage, but the Zn taken up by IR69428 accumulated in roots rather than being transported to grains. The findings of this paper would help in identifying donors for location specific breeding for Zn deficiency tolerance and Zn biofortication and thereby further crop improvement.

## Author contributions

Sarah E. Johnson-Beebout, Somayanda M. Impa, Anja Gramlich, Rainer Schulin, and Susan Tandy planned the experiments. Somayanda M. Impa and Anja Gramlich carried out the experiments under the supervision of Rainer Schulin and Emmanuel Frossard. Somayanda M. Impa and Sarah E. Johnson-Beebout wrote the paper with inputs from Rainer Schulin, Anja Gramlich, Susan Tandy, and Emmanuel Frossard.

### Conflict of interest statement

The authors declare that the research was conducted in the absence of any commercial or financial relationships that could be construed as a potential conflict of interest.

## Appendix

**Table A1:** **Growth parameters of unlabeled rice genotypes at different growth stages and Zn treatments in experiment-1**.

**Growth stages**	**Genotype**	**Treatment**	**Plant height (cm)**	**Root length (cm)**	**Shoot dry weight (g plant^−**1**^)**	**Root dry (g plant^−**1**^)**	**Total dry matter (g plant^−**1**^)**
21 DAP	IR55179	Zn-sufficient	50^b^ ± 1.6	22^b^ ± 0.7	1.1^b^ ± 0.14	0.5^b^ ± 0.05	1.6^b^ ± 0.18
		Zn-deficient	40^c^ ± 0.6	18^c^ ± 0.6	0.7^c^ ± 0.05	0.3^c^ ± 0.03	1.0^c^ ± 0.07
	KP	Zn-sufficient	68^a^ ± 1.2	26^a^ ± 0.5	1.6^a^ ± 0.09	0.6^a^ ± 0.04	2.1^a^ ± 0.12
		Zn-deficient	52^b^ ± 1.0	23^b^ ± 0.7	0.8^c^ ± 0.04	0.3^c^ ± 0.02	1.0^c^ ± 0.06
	Genotype		*p* < 0.001	*p* < 0.001	*p* = 0.002	*p* = 0.05	*p* = 0.005
	Treatment		*p* < 0.001	*p* < 0.001	*p* < 0.001	*p* < 0.001	*p* < 0.001
	Genotype × treatment		*p* = 0.01	NS	NS	NS	NS
	5% LSD (Zn × G)		3	2	0.2	0.1	0.3
42 DAP	IR55179	Zn-sufficient	54^b^ ± 0.3	34^a^ ± 0.6	4.8^b^ ± 0.2	2.4^a^ ± 0.06	7.2^b^ ± 0.2
		Zn-deficient	39^c^ ± 1.2	17^b^ ± 1.2	2.7^c^ ± 0.1	0.2^c^ ± 0.02	2.9^c^ ± 0.1
	KP	Zn-sufficient	72^a^ ± 2.0	43^a^ ± 5.2	6.9^a^ ± 0.2	2.4^a^ ± 0.08	9.3^a^ ± 0.2
		Zn-deficient	54^b^ ± 2.5	40^a^ ± 0.7	1.3^d^ ± 0.2	0.6^b^ ± 0.08	1.9^d^ ± 0.2
	Genotype		*p* < 0.001	*p* = 0.001	*p* = 0.04	*p* = 0.02	*p* = 0.008
	Treatment		*p* < 0.001	*p* = 0.009	*p* < 0.001	*p* < 0.001	*p* < 0.001
	Genotype × treatment		NS	*p* = 0.05	*p* < 0.001	NS	*p* < 0.001
	5% LSD (Zn × G)		6	10	0.5	0.3	0.5

DAP, days after planting in ANS. Zn × G, Zn treatment × genotype. Values with different letters in a column within a growth stage are significantly different at 5% LSD. Shoot = leaf lamina + sheath + stem. The values given are means ± SE (n = 10 for 21 DAP and n = 3 for 42 DAP).

**Table A2:** **Growth parameters of unlabeled rice genotypes in Zn-sufficient conditions in experiment-3**.

**Trait**	**Ten days after 50% flowering**		**Maturity**	
	**SWHOO**	**IR69428**	**SWHOO**	**IR69428**
Plant height (cm)	77 ± 3	78 ± 6	80 ± 3	79 ± 3
Root length (cm)	43 ± 2	48 ± 2	41 ± 0.9	54 ± 5
Tiller number (plant^−1^)	8 ± 0.7	4 ± 0.4	6 ± 1	5 ± 0.6
Root dry weight (g plant^−1^)	4 ± 1	4 ± 0.8	5 ± 1	4 ± 1.6
Stem dry weight (g plant^−1^)	12 ± 0.6	16.4 ± 2	13 ± 1	19 ± 0.4
Dry leaf dry weight (g plant^−1^)	–	–	1 ± 0.3	4 ± 0.2
Green leaf dry weight (g plant^−1^)	4 ± 0.4	6 ± 0.7	2 ± 0.5	2 ± 0.8
Panicle dry weight (g plant^−1^)	3 ± 0.7	2 ± 0.1	–	–
Rachis dry weight (g plant^−1^)	–	–	0.3 ± 0.03	0.3 ± 0.06
Hull dry wieght (g plant^−1^)	–	–	1 ± 0.2	0.5 ± 0.09
Apical grain weight (g plant^−1^)	–	–	3 ± 0.5	2 ± 0.2
Distal grain weight (g plant^−1^)	–	–	3 ± 0.4	2 ± 0.04
